# Hispidulin Alleviates Mast Cell-Mediated Allergic Airway Inflammation through FcεR1 and Nrf2/HO-1 Signaling Pathway

**DOI:** 10.3390/antiox13050528

**Published:** 2024-04-26

**Authors:** Seungwon Jeong, Yeon-Yong Kim, Dongwon Lee, Sang-Hyun Kim, Soyoung Lee

**Affiliations:** 1 Functional Biomaterial Research Center, Korea Research Institute of Bioscience and Biotechnology (KRIBB), 181, Ipsin-gil, Jeongeup 56212, Republic of Korea; jsw0212@kribb.re.kr (S.J.); gyy123@kribb.re.kr (Y.-Y.K.); 2 Department of Bionanotechnology and Bioconvergence Engineering, Jeonbuk National University, 567, Baekje-daero, Jeonju 54896, Republic of Korea; dlee@jbnu.ac.kr; 3 Department of Polymer Nano Science and Technology, Jeonbuk National University, 567, Baekje-daero, Jeonju 54896, Republic of Korea; 4 Cell Matrix Research Institute, Department of Pharmacology, School of Medicine, Kyungpook National University, Daegu 41944, Republic of Korea

**Keywords:** hispidulin, allergic asthma, airway inflammation, mast cell, oxidative stress

## Abstract

Allergic asthma is a type 2 immune-response-mediated chronic respiratory disease. Mast cell activation influences the pathogenesis and exacerbation of allergic asthma. Therefore, the development of mast cell-targeting pharmacotherapy is important for managing allergic airway inflammation. We investigated the efficacy of hispidulin (HPD), natural flavone, in a mast-cell-mediated ovalbumin (OVA)-induced allergic airway inflammation model. HPD alleviated symptoms of allergic asthma and decreased the levels of immunoglobulin (Ig) E, type 2 inflammation, immune cell infiltration, and mast cell activation in the lung. Furthermore, in vivo analysis confirmed the efficacy of HPD through the evaluation of IgE-mediated allergic responses in a mast cell line. HPD treatment inhibited mast cell degranulation through inhibition of the FcεR1 signaling pathway and suppressed the expression of inflammatory cytokines (TNF-α, IL-4, IL-6, and IL-13) through suppression of the NF-κB signaling pathway. The antioxidant effects of HPD in activated mast cells were identified through modulation of antioxidant enzymes and the Nrf2/HO-1 signaling pathway. In conclusion, HPD may be a potential therapeutic candidate for allergic airway inflammation of asthma and acts by suppressing mast cell activation and oxidative stress.

## 1. Introduction

Asthma is a chronic respiratory disease that affects approximately 260 million people worldwide, and the majority have allergic asthma (~80% and >50% of pediatric and adult patients with asthma, respectively) [1,2]. Allergic asthma can occur easily with exposure to various environmental factors, including food, pollen, dust mite, or dander. Continuous exposure to allergens triggers excessive airway inflammation and an exaggerated immune response. Airway inflammation is characterized by the recruitment of immune cells, including eosinophils, lymphocytes, neutrophils, and mast cells to the airway [3]. These cells release inflammatory mediators that promote bronchoconstriction, airway hyperresponsiveness, and mucus production [4]. These symptoms not only make breathing difficult but also cause irreversible respiratory damage. Another major feature of the airway inflammation induced in allergic asthma is a type 2 immune response that involves the release of various cytokines, including IL-4, IL-5, and IL-13 [5]. These cytokines promote immunoglobulin (Ig) E antibody production, eosinophil migration, and goblet cell hyperplasia, which exacerbate the symptoms of allergic asthma [6,7,8].

Mast cells, which are derived from the myeloid lineage, are innate immune cells that play a key role in allergic diseases, such as anaphylaxis, asthma, and atopic dermatitis [9]. Mast cells characteristically contain many granules involved in histamine release and induce the expression of surface receptors (FcεR1) that have a high affinity for IgE. During an allergic reaction, exposure to an allergen induces the formation of an allergen–IgE complex on the surface of mast cells through cross-linking of allergen IgE–FcεR1 [10] that activates Lyn, a member of the Src family tyrosine kinase. Activated Lyn phosphorylates splenic tyrosine kinase (Syk), which leads to the activation of various downstream signaling pathways, such as the phospholipase Cγ (PLC-γ) pathway [11]. This leads to the release of intracellular calcium and the activation of various transcription factors, which induce mast cell degranulation and release of various inflammatory mediators, such as histamine, leukotrienes, and inflammatory cytokines [11,12]. These mechanisms further intensify the inflammatory response and induce exacerbation of allergic inflammatory disease.

Oxidative stress plays an important role in the pathogenesis of various allergic and immunological diseases [13]. Levels of reactive oxygen species (ROS), including hydrogen peroxide and nitric oxide, are increased in patients with asthma [14]. In asthma, endogenous or exogenous ROS induction affects airway inflammation and contributes to the severity of asthma [15,16]. Excessive oxidative stress induces diverse proinflammatory mediators that aggravate asthmatic symptoms, including airway inflammation, hyper-responsiveness, and increased mucus secretion [17,18]. Although many studies have reported the significance of oxidative stress on the development and deterioration of airway inflammation, antioxidant therapy has been used as adjuvant therapy for steroid administration in allergic airway inflammation [19,20].

Hispidulin (5,7-dihydroxy-2-(4-hydroxyphenyl)-6-methoxy-4H-1-benzopyran-4-one, HPD), a type of naturally occurring flavone, is an organic compound that is derived from plants, such as Grindelia herb, Salvia, and Artemisia species, and is characteristically a yellowish powder. By acting as a benzodiazepine receptor ligand, HPD has been proven to have antioxidant, antifungal, anticonvulsive, and antithrombotic effects [21]. Recent studies have demonstrated the efficacy of HPD in various allergic disease models, such as anaphylaxis and house-dust-mite-induced atopic dermatitis [22,23]. However, the mechanism of action underlying HPD-based targeting of mast cells remains insufficiently elucidated, and the efficacy of HPD in allergic lung diseases has not been proven.

Based on a framework of the results from previous studies, we investigated whether HPD, in allergic asthma and acute lung injury models, is an effective treatment strategy for mast-cell-mediated allergic inflammatory diseases. Therefore, we examined the efficacy of HPD on ovalbumin (OVA)-induced allergic asthma model. Furthermore, using a mammalian cell line, we studied the effects of HPD on IgE-mediated allergic reactions in mast cells and elucidated the efficacy of HPD on the cellular and molecular mechanisms underlying mast cell-based reactions.

## 2. Materials and Methods

### 2.1. Materials

Hispidulin and dexamethasone were purchased from Sigma-Aldrich (St. Louis, MO, USA). To compare the efficacy of asthma treatment, dexamethasone (Dex) was used as the reference drug. Anti-dinitrophenyl (DNP) IgE, DNP-human serum albumin (HSA), *o*-phthaldialdehyde, and ovalbumin (OVA) were purchased from Sigma-Aldrich, whereas alum was provided by Thermo Fisher Scientific (Waltham, MA, USA). The enzyme-linked immunosorbent assay (ELISA) kits for mouse IL-4, IL-5, IL-6, and IL-13, for rat tumor necrosis factor (TNF)-α and IL-4, and for eosinophil peroxidase (EPO) were purchased from BD Biosciences, Invitrogen, and Ebioscience (San Diego, CA, USA), respectively.

### 2.2. Animals

To induce allergic asthma and develop an acute lung injury model, 6-week-old female BALB/c and ICR mice, respectively, were purchased from the Orent Bio (Gwangju, Republic of Korea) and used. All mice were fed a standard chow diet and filtered water during the study. The mice were housed 5 per cage, each of which had an individual ventilation system. All mice were housed in specific pathogen-free conditions and were bred in an environment with a constant light/dark 12 h cycle, appropriate temperature (22 ± 2 °C), and relative humidity (55 ± 5%). The animal experiments were approved by the Institutional Animal Care and Use Committee of the Korea Research Institute of Bioscience and Biotechnology (KRIBB-AEC-22021) and were carried out in compliance with the guidelines established by the Public Health Service Policy on the Humane Care and Use of Laboratory Animals. The animal experiments were conducted after a sufficient adaptation period.

### 2.3. Ovalbumin (OVA)-Induced Allergic Asthma Model

The BALB/c mice were randomly divided into five groups (*n* = 5) as follows: control, OVA, OVA + HPD 1 mg/kg (low-dose), OVA + HPD 10 mg/kg (high-dose), and OVA + Dex 2 mg/kg (positive control drug). The mice were intraperitoneally sensitized with the OVA mixture (OVA 100 μg + alum 4 mg in PBS 200 μL) on days 0 and 7. Then, the mice were challenged for 30 min with OVA 3% solution in PBS that was administered as an aerosol by using an ultrasonic nebulizer (NE-U17; OMRON Corp., Tokyo, Japan) on days 14–17. At the time of the challenge, the mice received daily oral administration of PBS, HPD, or Dex. On Day 18, all mice were euthanized via isoflurane inhalation, and blood, bronchoalveolar lavage fluid (BALF), lung, spleen, and lymph node samples were collected for analysis (Figure S1). The splenic weight was measured immediately after sacrifice; the BALF sample was centrifuged at 500× *g* for 5 min at 4 °C, and the supernatant was stored at −80 °C.

### 2.4. Lipopolysaccharide (LPS)-Induced Acute Lung Injury Model

The ICR mice were randomly divided into five groups (*n* = 5) as follows: control, LPS, LPS + HPD 1 mg/kg (low-dose), LPS + HPD 10 mg/kg (high-dose), and LPS + Dex 2 mg/kg (positive control drug). The mice were treated with orally administered PBS, HPD, or Dex. After 1 h, the LPS group was administered 1 mg/mL of LPS solution through tracheal injection (injection volume: 25 μL), and control mice received 25 μL PBS. Subsequently, all mice were euthanized with isoflurane inhalation, and samples of blood, BALF, and lung were collected for analysis (Figure S2). The BALF sample was centrifuged at 500× *g* for 5 min at 4 °C, and the supernatant was stored at −80 °C.

### 2.5. Cell Culture

Rat basophilic leukemia (RBL)-2H3 and the human mast cell line (HMC-1) were purchased from ATCC (Manassas, VA, USA) and Sigma-Aldrich. The RBL-2H3 and HMC-1 cell line were cultured in Dulbecco’s Modified Eagle’s medium (DMEM, GIBCO, Grand Island, NY, USA) and Iscove’s Modified Dulbecco’s Medium (IMDM, GIBCO), respectively, and then supplemented with heat-inactivated 10% fetal bovine serum (FBS, GIBCO) and 1% antibiotic–antimycotic (comprising 10,000 units/mL penicillin, 25 μg/mL amphotericin B, and 10,000 μg/mL streptomycin, GIBCO). Cells were incubated at 37 °C in 5% CO_2_. The cells of passages 5–8 were used in this study.

### 2.6. Cytotoxicity

The viability of RBL-2H3 and HMC-1 was assayed using an MTT (Invitrogen, Inc.) and EZ-Cytox (Water-Soluble Tetrazolium (WST)-1 assay reagent (Dogen, Seoul, Republic of Korea)). RBL-2H3 cells (4 × 10^4^ cells/well) were seeded in a 96-well plate and cultured at 37 °C for 18 h. Then, the RBL-2H3 cells were pretreated with various HPD concentrations for 24 h and incubated with 1 mg/mL MTT reagent at 37 °C for 2 h. After the supernatant was removed, 200 μL dimethyl sulfoxide (DMSO) was added to each well to dissolve formazan crystals in the cells. Similarly, the HMC-1 cells (4 × 10^4^ cells/well) were seeded in 96-well plate and incubated at 37 °C for 18 h, and were pretreated with various HPD concentrations for 24 h and incubated with 100 μL WST-1 reagent at 37 °C for 2 h. The absorbance was measured at 570 and 450 nm using a microplate reader (Thermo Fisher Scientific, Inc.). The cell viability was calculated as the relative absorbance ratio compared to the control.

### 2.7. Diff-Quik Staining

In centrifuged BALF, cell pellets were resuspended in PBS and centrifuged using cytospin before transfer onto a glass slide for Diff-Quik staining with a fixative reagent (Methanol), solution 1 (Eosinophilic, orange), and solution 2 (basophilic, deep blue). The slides were dipped into each solution 5 times sequentially and then rinsed with water and dried. After staining, immune cells were counted using a hemocytometer.

### 2.8. ELISA

The level of cytokines and inflammatory mediators in the biological fluids (BALF, serum Ig, and cell culture supernatant) were measured with ELISA by using the ELISA kit in a 96-well Nunc-immune plate according to the manufacturer’s protocol. For OVA-specific-IgE, OVA solution (10 μg/mL), and not the extracted antibodies, was directly coated on the immune plate. For the cell-culture supernatant, RBL-2H3 (5 × 10^5^ cells/well in 12-well plate) sensitive to anti-DNP IgE (100 ng/mL) was pretreated with HPD for 1 h (after washing 3 times with PBS) and stimulated with DNP-HSA (100 ng/mL) for 6 h. Absorbance was measured at 450 nm using a microplate reader. The quantitative value of each cytokine and enzyme was quantified according to the standard curve, and the OVA-specific IgE levels were expressed as the optical density (O.D) value.

### 2.9. Quantitative Polymerase Chain Reaction (qPCR)

The expression of inflammatory cytokines in the lung and lymphatic tissues of OVA-induced allergic asthma model mice and RBL-2H3 cells challenged with DNP-HSA were measured by qPCR. After euthanasia, the lung and lymphatic tissues of mice were collected, and immediately stored at −80 °C in RNAlater (Invitrogen, Inc.). For RNA extraction, TRIzol Reagent (Invitrogen) was used as follows: each tissue sample was homogenized in 700 μL TRIzol Reagent. Then, 100 μL chloroform was added and weakly vortexed for 10 s, 3 times, and incubated at room temperature (RT) for 5 min and centrifuged at 13,000× *g* under 4 °C for 15 min. Next, 200 μL supernatant was transferred to a new 1.5-mL tube. The same volume of isopropanol was added, and the mixture was gently inverted 5–7 times and incubated for 5 min at RT. All samples were centrifuged at 13,000× *g* and 4 °C for 15 min and the supernatant was pipetted out. The RNA pellet was washed with 75% ethanol twice, the leftover ethanol was removed, and the RNA pellet was air-dried for 10 min and dissolved in nuclease-free water. Anti-DNP IgE (100 ng/mL)-sensitized RBL-2H3 (5 × 10^5^ cells/well in 12-well plates) were pretreated with HPD for 1 h and then stimulated with DNP-HSA (100 ng/mL) for 1 h. After the cell culture supernatant was removed, 500 μL TRIzol Reagent was added. The total cell RNA extraction was undertaken using the same procedure. The isolated RNA was quantified with a Micro-Spectrophotometer (Allsheng, Inc., Hangzhou, China) and referred to an A 260/A 280 ratio of 1.8–2.1. First-strand complementary DNA (cDNA) was synthesized using a Thermo cDNA synthesis kit (Thermo Fisher Scientific), and qPCR was performed using qTOWER (Analytik jena, Jena, Thuringia, Germany). Briefly, 1 μL cDNA, 0.5 μL each of forward and reverse primers (0.2 μM), 7.5 μL SYBR Premix Ex Taq (Takara Bio, Inc., Shiga, Japan), and 5.5 μL dH_2_O were mixed to obtain a 15-μL reaction mixture. The amplification conditions were 30 s at 95 °C, 40 cycles of 5 s at 95 °C, 5 s at 58 °C, and 15 s at 72 °C, followed by 6 s for melting. The relative quantification of mRNA expression was performed using the qPCRsoft 3.0 software (Analytik jena, Jena, Thuringia, Germany). The primers (sequences are shown in Table S1) were purchased from Bioneer (Daejeon, Republic of Korea). The cycle numbers were optimized to ensure product accumulation in the exponential range. β-actin was used as the normalization control.

### 2.10. β-Hexosaminidase Assay

The release of β-hexosaminidase in BALF and cell culture media was measured with a β-hexosaminidase assay. In animal studies, 40 μL BALF was transferred to 96-well plates and incubated with an equal volume of substrate solution (1 mM 4-nitrophenyl N-acetyl-β-D-glucosaminide in 0.1 M citrate buffer, pH 4.5) for 1 h at 37 °C. The reaction was terminated by adding 200 μL stop solution (0.1 M Na_2_CO_3_-NaHCO_3_, pH 10). In cell experiments, anti-DNP IgE (100 ng/mL)-sensitized RBL-2H3 cells (5 × 10^5^ cells/well in 12-well plates) were pretreated with HPD or Dex for 1 h. After washing with PBS 3 times, the sensitized RBL-2H3 cells were stimulated with DNP-HSA (100 ng/mL) for 4 h at 37 °C. The cell culture media was centrifuged at 150× *g* for 3 min, and the supernatant was transferred to a 1.5-mL tube. Adherent cells were then lysed with 0.5% Triton X-100. Similarly, the cell-lysed solution was centrifuged and transferred to a 1.5-mL tube. After 1 h pretreatment with HPD, HMC-1 cells (5 × 10^5^ cells/well in 12-well plates) were stimulated with phorbol 12-myristate 13-acetate (PMA) and A23187 for 6 h at 37 °C. After incubation, the cell culture media was centrifuged at 150× *g* for 3 min and the supernatant was transferred to a 1.5 mL tube. The cell pellet was lysed with 0.5% Triton X-100. The cell-lysed solution was centrifuged and transferred to a 1.5-mL tube. Next, 40 μL of each sample was transferred to a 96-well plate, and the enzyme–substrate reaction was undertaken as described earlier. The absorbance was measured at 405 nm using a microplate reader. The release of β-hexosaminidase in BALF and culture media was determined by the O.D value.

### 2.11. Histamine Assay

To measure histamine in cell culture media, 80 μL 0.1 N HCl and 20 μL 60% perchloric acid were added to a 200-μL sample. After vortexing for 30 s, the samples were centrifuged at 13,000× *g*, 4 °C for 20 min. The supernatant was transferred to 1.5-mL tubes; thereafter, 100 μL 5 N NaOH, 200 μL 5 M NaCl, and 800 μL n-butanol were added and vortexed for 30 s. The samples were then centrifuged. Next, 500 μL supernatant was mixed with 200 μL 0.1 N HCl and 500 μL n-heptane, centrifuged at 13,000× *g*, 4 °C, and 150 μL of the bottom layer (water layer) was transferred to a 96-well black plate. Then, 40 μL NaOH and 10 μL 1% *o*-phthaldialdehyde were added to induce a reaction with histamine. The fluorescence intensity was measured at Ex/Em 360/440 nm using a fluorescence plate reader (Molecular Devices, San Jose, CA, USA).

### 2.12. Intracellular Calcium Assay

The concentration of intracellular calcium was measured using the fluorescence indicator Fluo-4 AM (Invitrogen). Anti-DNP IgE (100 ng/mL)-sensitized RBL-2H3 (2 × 10^4^ cells/well in 96-well plates) were preincubated with Fluo-4 AM (5 μM) for 1 h at 37 °C. After washing 2 times with PBS, the cells were treated with HPD or BAPTA-AM for 1 h and stimulated with DNP-HSA (100 ng/mL). After DNP-HSA treatment, the intracellular calcium level was kinetically measured for total 150 s, every 30 s, 5 times. When the positive control group (DNP-HSA) showed the maximum value (120 s), it was designated as an endpoint and analysis was performed (Figure S3). BAPTA-AM, a calcium chelator, was used as a positive control. The fluorescence intensity was measured under Ex/Em 485/510 nm using a fluorescence plate reader (Molecular Devices).

### 2.13. Intracellular ROS Assay

The concentration of intracellular ROS was measured using the fluorescence indicator H_2_DCFDA (Invitrogen). Anti-DNP IgE (100 ng/mL)-sensitized RBL-2H3 (2 × 10^4^ cells/well in 96-well plates) were treated with HPD or Dex for 1 h and stimulated with DNP-HSA (100 ng/mL) for 4 h at 37 °C. After incubation, 100 μL DCF-DA solution (final concentration: 10 μM) was added and treated for 1 h at 37 °C. The fluorescence intensity was measured under Ex/Em 495/525 nm using a fluorescence plate reader (Molecular Devices).

### 2.14. Western Blotting

Anti-DNP IgE (100 ng/mL)-sensitized RBL-2H3 (1 × 10^6^ cells/well in 6-well plates) were pretreated with HPD for 1 h. After washing 3 times with PBS, sensitized RBL-2H3 cells were stimulated with DNP-HSA (100 ng/mL) for 7 min, 30 min, 1 h, and 3 h. The cells were then washed 2 times with PBS and lysed with 100 μL cell lysis buffer (Cell Signaling Technology, Danvers, MA, USA) containing 0.5 mM PMSF/DTT and 1 × Protease and Phosphatase Inhibitor Cocktail (Thermo Fisher Scientific). The lysates were vortexed, incubated for 30 min on ice, and centrifuged at 13,000× *g* for 30 min at 4 °C. In case of nuclear and cytosolic protein extraction, NE-PERTM Nuclear and Cytoplasmic Extraction Kit (Thermo Fisher Scientific) was used and each protein was extracted as described in the manufacturer’s protocol. The supernatants were collected and the total protein was quantified using Bio-Rad Protein Assay Dye Reagent Concentrate (Bio-Rad, Hercules, CA, USA) for the Bradford Assay. Equal amounts of protein lysate were loaded onto NuPAGE 10% Bis-Tris gel (Invitrogen) for electrophoresis. Next, the protein bands were transferred to a polyvinylidene difluoride (PVDF) membrane. After blocking with 5% bovine serum albumin (BSA) in TBST, the membrane was incubated with the target primary antibody, with shaking, at 4 °C overnight. After washing 3 times with TBST (Tris-buffered saline with 0.1% Tween 20) each for 10 min, the PVDF membrane was incubated with anti-IgG horseradish peroxidase-conjugated secondary antibody at RT for 1 h. After washing 3 times, immunoreactive protein bands were visualized using a Miracle-Star Western blot detection system (iNtron Biotechnology, Seongnam, Korea) and SuperSignal™ West Femto Maximum Sensitivity Substrate (Thermo Fisher Scientific), and the results were analyzed using ChemiDoc XRS+ System (Bio-rad). The antibodies (listed in Table S2) were purchased from Cell Signaling Technology (CST).

### 2.15. Statistical Analysis

Data were analyzed using GraphPad Prism version 7 (GraphPad Software, La Jolla, CA, USA). The treatment effects were analyzed using one-way analysis of variance followed by Dunnett’s test. Statistical significance was set at *p* < 0.05.

## 3. Results

### 3.1. HPD Attenuates OVA-Induced Allergic Asthma

**Figure 1:**
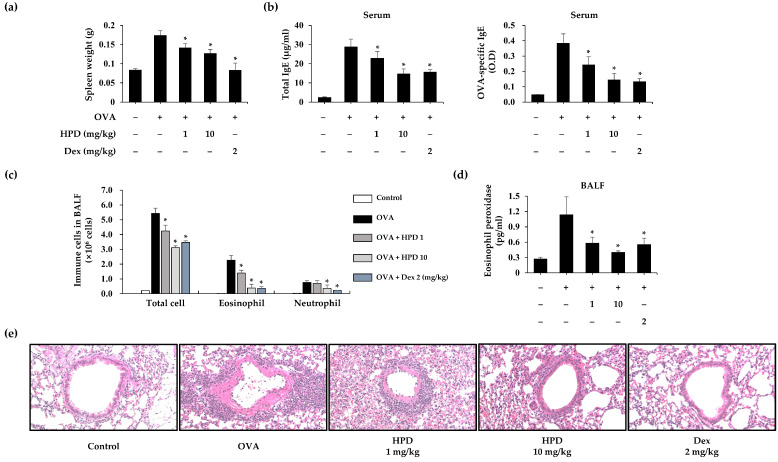
Effects of HPD on immunoglobulin production and immune cells infiltration and activation in OVA-induced allergic asthma mice. (**a**) After mice were sacrificed, spleen weight was measured. (**b**) The levels of immunoglobulin (total and OVA-specific IgE) was measured by ELISA. (**c**) After total cells in BALF were measured by cell counter, each immune cell was stained with Diff-Quik. (**d**) The eosinophil peroxidase levels were measured by ELISA. Values are mean ± SEM (*n* = 5). * *p* < 0.05 relative to OVA group. (**e**) The paraffin-embedded lung sections were stained with hematoxylin and eosin. Dex: dexamethasone. The magnification of representative images was 200×.

To investigate the efficacy of HPD on allergic asthma, an OVA-induced asthma model was established (Figure S1). In allergic reactions, splenomegaly is one of the main characteristics of an exaggerated immune reaction. To confirm the mitigation of the immune response, we first measured the variations in splenic weight after the mice were euthanized and found that HPD-treated mice had a diminished increase in splenic weight (Figure 1a). IgE secreted by activated B cells mediated the allergic reaction. To assess the effect of HPD on IgE production, the serum levels of total and OVA-specific IgE were measured, which showed elevated levels in the OVA-treated group, whereas HPD inhibited the secretion of total and OVA-specific IgE (Figure 1b). The major symptoms of asthma-induced lung injury are the recruitment and infiltration of immune cells, which release inflammatory mediators that exacerbate allergic asthma. To assess the impact of HPD on the asthmatic lung, we analyzed the BALF samples from the allergic asthma model and found that the number of various immune cells, such as eosinophil and neutrophil, had decreased in the HPD-treated group (Figure 1c). Furthermore, we quantified the level of inflammatory enzymes, including eosinophil peroxidase (EPO), which are released by activated eosinophils. The results indicated that EPO release in BALF was alleviated by HPD treatment (Figure 1d). Next, the structural changes in lung sections were histopathologically confirmed through Hematoxylin and Eosin (H&E) staining. The infiltration of immune cells increased in the OVA-treated group but decreased in the HPD-treated group (Figure 1e). These results suggest that HPD alleviates OVA-induced allergic asthma. Dexamethasone (Dex), a corticosteroid that is widely used in the clinical treatment of allergies and other pulmonary diseases, was used as a positive control.

### 3.2. HPD Attenuates Airway Inflammation by Suppressing Type 2 Immune Response in OVA-Induced Allergic Asthma

**Figure 2:**
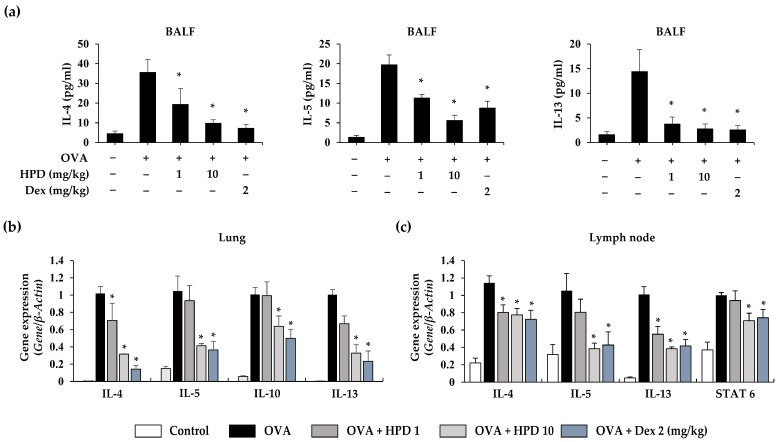
Effects of HPD on type 2 inflammation in OVA-induced allergic asthma. (**a**) The levels of type 2 cytokines (IL-4, IL-5 and IL-13) was measured by ELISA. (**b**,**c**) In tissue homogenates, the gene expression levels in lung (IL-4, IL-5, IL-10 and IL-13) and lymph node (IL-4, IL-5, IL-13 and STAT6) were analyzed by qPCR. Dex: dexamethasone. Values are mean ± SEM (*n* = 5). * *p* < 0.05 relative to OVA group.

Allergic asthma predominantly involves a type 2 immune response that is mediated by type 2 cytokines. These cytokines cause pathological changes at the inflammatory site to induce exacerbation of asthmatic symptoms. To investigate the efficacy of HPD on allergic airway inflammation, BALF and lung tissue samples were analyzed by ELISA and qPCR. We evaluated the level of cytokines, such as IL-4, IL-5, and IL-13, and confirmed the production of type 2 cytokines (IL-4, IL-5, and IL-13) in BALF. HPD treatment alleviated the production of these inflammatory cytokines (Figure 2a). Next, we evaluated the gene expression of type 2 inflammatory cytokines (IL-4, IL-5, IL-10, and IL-13), which increased in lungs exposed to OVA but were suppressed with HPD treatment (Figure 2b). Moreover, cytokine expression in lymph nodes was evaluated because lymph nodes play an important role through T-cell activation-based mediation of the immune response to allergic asthma. We evaluated the gene-based expression of type 2 cytokines (IL-4, IL-5, and IL-13) and STAT 6, which is a transcription factor that plays a crucial role in the pathogenesis of allergen-induced airway inflammation. The gene expression levels were increased in OVA-exposed mice, whereas they was suppressed after HPD treatment (Figure 2c). These results suggest that HPD alleviates airway inflammation by suppressing the type 2 immune response in OVA-induced allergic asthma.

### 3.3. HPD Suppresses Mast-Cell-Mediated Immune Response in Allergic Asthma

**Figure 3:**
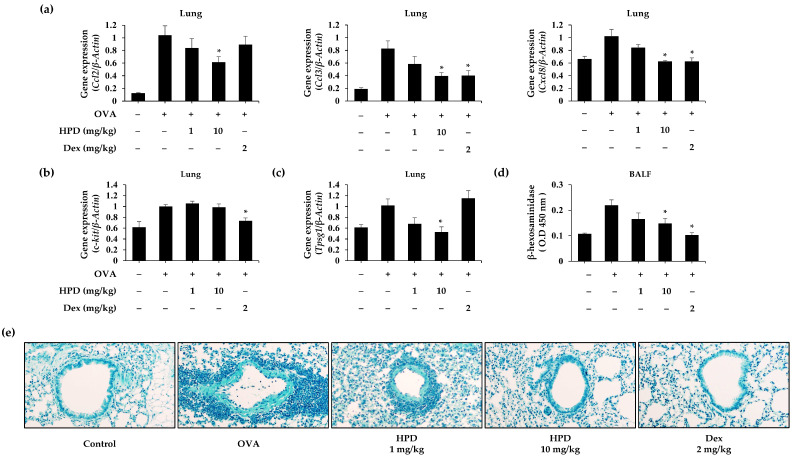
Effects of HPD on mast cell mediated immune response in OVA-induced allergic asthma. (**a**) The gene expression of chemokines (CCL2, CCL3, and CXCL8) was analyzed by qPCR. (**b**,**c**) The gene expression of mast cell markers (c-kit and Tpsg1) was analyzed by qPCR. β-Actin was used as internal control to normalize gene. (**d**) The degranulation of mast cells was measured by β-hexosaminidase assay. Values are mean ± SEM (*n* = 5). * *p* < 0.05 relative to OVA group. The paraffin-embedded lung sections were stained with Toluidine blue (**e**). Dex: dexamethasone. The magnification of representative images was 200×.

In allergic asthma, the recruitment of immune cells to the inflammatory site is a key feature. Mast cells, as effector cells, play a significant role in the immune response during allergic reactions. To investigate that the effect of HPD on mast-cell-mediated immune response in OVA-induced airway inflammation, we assessed the gene expression of chemokines (CCL2, CCL3, and CXCL8) released in activated mast cells using qPCR. The results revealed increased gene expression of chemokines in the OVA-treated group, while HPD treatment effectively suppressed these chemokines in the lung (Figure 3a). Furthermore, we examined the gene expression of c-kit and tryptase gamma-1 (Tpsg1), recognized as mast cell-specific markers. The findings indicated that HPD did not suppress the expression of c-kit in the OVA-induced asthmatic lung, but it effectively suppressed the expression of Tpsg1 (Figure 3b,c). Additionally, we analyzed the release of β-hexosaminidase, a biomarker released during mast cell degranulation. The results demonstrated increased β-hexosaminidase activity in OVA-induced allergic asthma, while HPD treatment reduced this enzyme activity (Figure 3d). Furthermore, we assessed mast cell infiltration into lung tissue using toluidine blue staining to visualize mast cell granules. Our results showed that HPD treatment reduced mast cell infiltration in the OVA-induced asthmatic lung (Figure 3e). These findings suggest that while HPD may not effectively differentiate mast cells, it does successfully suppress mast cell activation and infiltration in allergic airway asthma.

### 3.4. HPD Suppresses Mast Cell Degranulation In Vitro Assay

**Figure 4:**
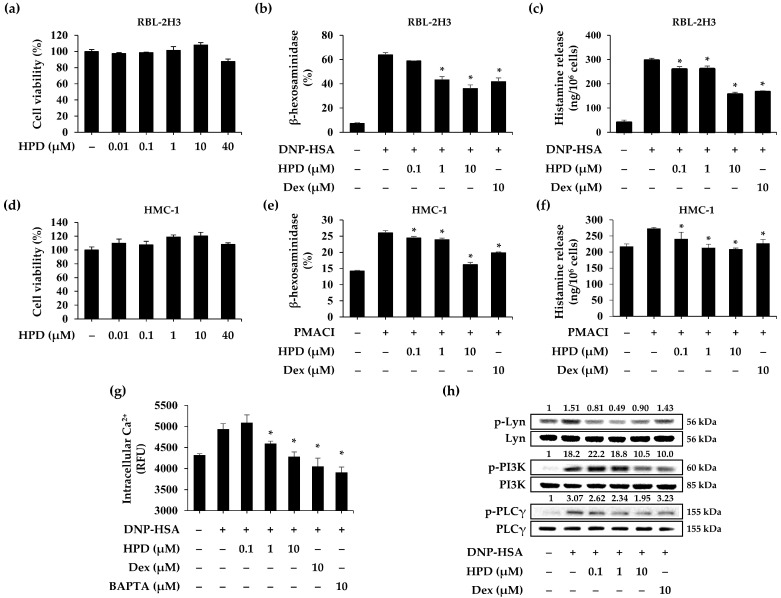
Effects of HPD on mast cell degranulation and signaling pathway. RBL-2H3 and HMC-1 cells were incubated at different concentrations (0.1–40 μM). After 24 h, each cell viability was measured by (**a**) MTT and (**d**) WST-1 assay. Values are mean ± SEM (*n* = 5). The degranulation of mast cells was measured by (**b**,**e**) β-hexosaminidase and (**c**,**f**) histamine assay using conditioned media of RBL-2H3 and HMC-1 cells. Values are mean ± SEM (*n* = 3). (**g**) Intracellular calcium level was measured by using Fluo-4 AM in RBL-2H3 cells. Values are mean ± SEM (*n* = 5). (**h**) The phosphorylation of Lyn, PI3K, and PLC-γ was measured by Western blot. The total forms of each protein were used as loading controls. Dex: dexamethasone. * *p* < 0.05 relative to DNP-HSA or PMACI group.

Based on the results of animal study, the RBL-2H3 and HMC-1 cell lines were used to confirm the more fundamental effect of HPD. RBL-2H3 cell line, a rat basophilic leukemia cell line with numerous IgE receptors in the cell membrane and similar structural function as mast cells, is commonly used for mast cell research. The cytotoxicity of HPD on mast cells was evaluated in the RBL-2H3 and HMC-1 cell lines by using the MTT and WST-1 assays. The results showed no confirmed cytotoxicity up to 40 μM HPD (Figure 4a,d). Subsequently, we investigated the efficacy of HPD in activated mast cells. To evaluate the effect of HPD on mast cell degranulation, we analyzed the level of β-hexosaminidase and histamine in the supernatant of activated cells, respectively. The DNP-IgE-sensitized RBL-2H3 cells were challenged with DNP-HSA and released high levels of β-hexosaminidases and histamine. However, the release of β-hexosaminidase and histamine in HPD treatment was alleviated in a concentration-dependent manner (Figure 4b,c). PMA and calcium ionophore (A23187) were used to stimulate HMC-1 cells, and PMACI-stimulated HMC-1 cells released β-hexosaminidase and histamine. Nevertheless, HPD treatment alleviated the release of β-hexosaminidase and histamine (Figure 4e,f). Calcium ions (Ca^2+^) are a crucial regulator of mast cell degranulation. To evaluate the calcium release in activated mast cells, we used Fluo-4 AM, a fluorescent dye reacting with calcium, to measure the intracellular calcium level in DNP-HSA-stimulated RBL-2H3 cells. The results showed that HPD treatment effectively alleviated intracellular calcium in activated RBL-2H3 cells (Figure 4g). Furthermore, to further evaluate the effect of HPD on the molecular signaling pathway in mast cell degranulation, we assessed the efficacy of HPD for the activation of Lyn, PI3K, and PLCγ in challenged RBL-2H3 cells. The results showed that HPD treatment in challenged RBL-2H3 cells suppressed phosphorylation of the downstream signaling pathway from Lyn to PLCγ (Figure 4h). Thus, these findings suggest that HPD inhibits degranulation of activated mast cells.

### 3.5. HPD Suppresses the Inflammatory Response for Mast Cell Activation

**Figure 5:**
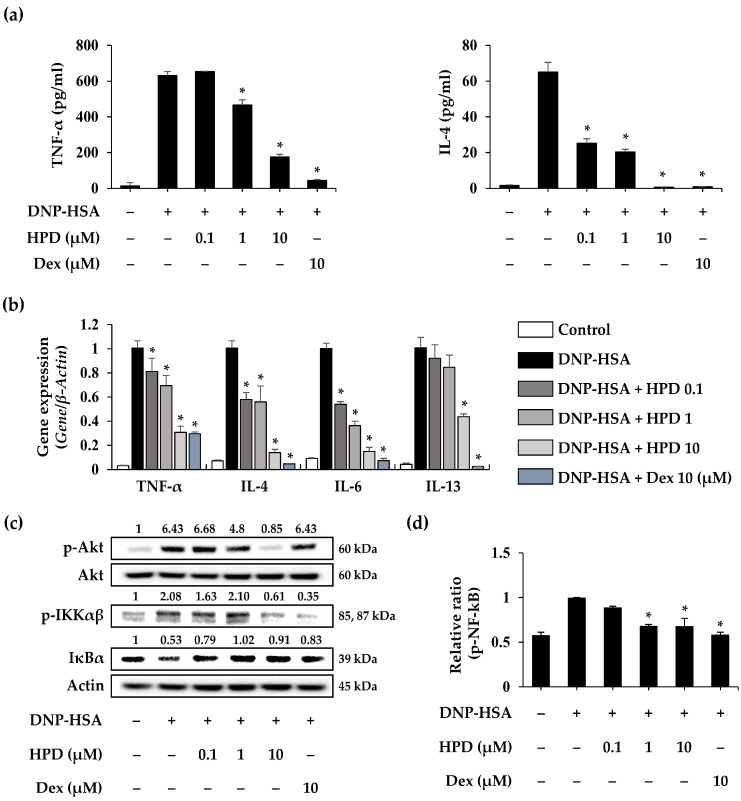
Effects of HPD on inflammatory cytokine secretion and expression in activated mast cells. (**a**) The levels of cytokines (TNF-α and IL-4) were measured by ELISA. (**b**) The gene expression of cytokines was measured by qPCR. β-Actin was used as internal control to normalize genes. (**c**) The p-Akt, p-IKKαβ, and IκBα were measured by Western blot. The actin was used as a loading control. (**d**) The level of p-NF-κB was measured by ELISA. Dex: dexamethasone. Values are mean ± SEM (*n* = 3). * *p* < 0.05 relative to DNP-HSA group.

Activated mast cells release various inflammatory cytokines and mediators induced by degranulation. To evaluate the effect of HPD on activated mast cells, we used ELISA assay and qPCR to analyze cytokine production and gene expression in challenged RBL-2H3 cells. In the cell supernatants, levels of cytokines, including TNF-α and IL-4, increased in DNP-HSA-stimulated RBL-2H3 cells. However, HPD treatment induced a concentration-dependent reduction in TNF-α and IL-4 secretion (Figure 5a). Subsequently, we validated the gene expression in DNP-HSA-stimulated RBL-2H3 cells and found that HPD treatment reduced gene expression, including that of TNF-α, IL-4, IL-6, and IL-13 (Figure 5b). To further evaluate the effect of HPD on the molecular signaling pathway, Western blot analysis was conducted. The results demonstrated that HPD inhibited several steps in the signaling pathway associated with mast cell activation, ranging from Akt phosphorylation to NF-κB (Figure 5c,d). Furthermore, total IκBα decreased in challenged RBL-2H3 cells, whereas HPD treatment increased IκBα (Figure 5c). These findings suggest that HPD inhibits the inflammatory response of activated mast cells by modulating the Akt-mediated NF-κB signaling pathway.

### 3.6. HPD Alleviates Nrf2/HO-1 Pathway Activation through Modulation of Antioxidant Enzyme Expression

**Figure 6:**
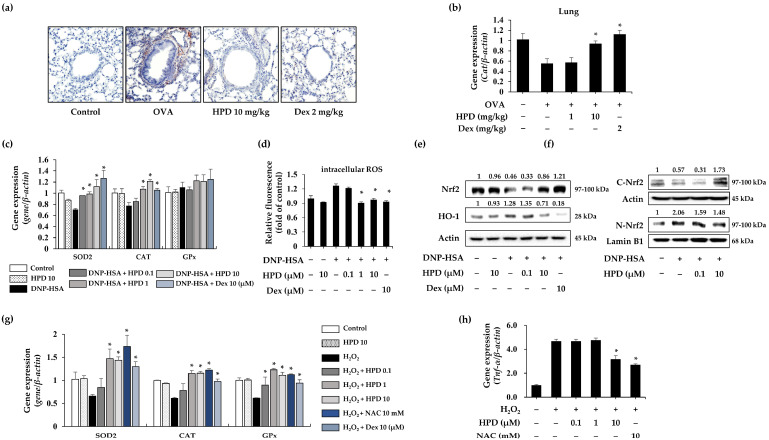
Antioxidant effects of HPD on OVA-induced mice and mast cells stimulated with DNP-HSA or H_2_O_2_. (**a**) The paraffin-embedded lung sections were stained with iNOS antibody. (**b**) The gene expression of catalase in lung homogenates of OVA-induced mice were measured by qPCR. Values are mean ± SEM (*n* = 5). * *p* < 0.05 relative to OVA. (**c**) The gene expression of antioxidant enzymes (SOD2, CAT, and GPx) in DNP-has-stimulated RBL-2H3 cell was analyzed by qPCR. (**d**) Relative intracellular ROS level was measured by using H_2_DCF-DA. (**e**) The Nrf2 and HO-1 in whole cell lysates were measured by Western blot. (**f**) The cytoplasmic and nuclear Nrf2 were measured by Western blot. The actin and Lamin B1 were used as a loading control. (**g**) The gene expression of antioxidant enzymes (SOD2, CAT, and GPx) in H_2_O_2_-stimulated RBL-2H3 cell was analyzed by qPCR. (**h**) The TNF-α expression in H_2_O_2_-stimulated RBL-2H3 was analyzed by qPCR. Dex: dexamethasone. NAC: N-acetylcysteine. Values are mean ± SEM (*n* = 3). * *p* < 0.05 relative to DNP-HSA or H_2_O_2_ group.

Oxidative stress is a critical factor influencing the development of inflammation, and dysregulation of nitric oxide production has been reported in asthma [13]. Using immunohistochemistry (IHC), we examined the expression of inducible nitric oxide synthase (iNOS), a nitric oxide-producing enzyme [24]. The iNOS levels increased in OVA-induced lung tissue sections were suppressed by HPD treatment (Figure 6a). Moreover, we investigated the gene expression of the antioxidant enzyme catalase in asthmatic lung tissue using qPCR. The results showed that the diminished gene expression of catalase in the asthmatic lung was restored through HPD treatment (Figure 6b). To evaluate the antioxidant effect of HPD on mast cell activation, we analyzed the expression change of antioxidant genes (SOD2, CAT, and GPx) in challenged RBL-2H3 cells. DNP-HSA stimulation led to a decrease in the expression of SOD2 and CAT, but not GPx. However, HPD treatment not only alleviated the expression of antioxidant genes, including SOD2 and CAT, but also increased CAT more than in the control group. Notably, HPD was less effective against GPx (Figure 6c). We assessed the effect of HPD on relative intracellular ROS in challenged RBL-2H3 cells using the fluorescence dye H_2_DCF-DA. The results revealed an increase in intracellular ROS levels in DNP-HSA-challenged RBL-2H3 cells, whereas HPD treatment effectively reduced intracellular ROS levels in activated RBL-2H3 cells (Figure 6d). We next investigated the effects of HPD on the Nrf2/HO-1 signaling pathway in mast cell activation. The results showed that total Nrf2 levels in DNP-HSA-stimulated RBL-2H3 cells decreased in whole-cell lysate, but HPD treatment enhanced Nrf2 levels. However, despite the fact that HO-1 levels in activated mast cells were increased, HPD treatment led to a decrease in its levels in whole-cell lysate (Figure 6e). To further confirm the reduction of HO-1 expression by HPD treatment, we identified nuclear-translocated Nrf2. HPD treatment in DNP-HSA-stimulated RBL-2H3 cells reduced the nuclear translocation of Nrf2 (Figure 6f). We established that mast cell activation induces an imbalance in antioxidant genes, leading to the accumulation of intracellular oxidative stress. To evaluate the effect of HPD in conditions of excessive oxidative, we analyzed the expression of antioxidant genes (SOD2, CAT, and GPx) in H_2_O_2_-treated RBL-2H3 cells. The results showed that H_2_O_2_ stimulation reduced gene expression, whereas HPD treatment not only restored gene expression but also increased it further than in the control group (Figure 6g). Furthermore, to evaluate the effect of HPD on proinflammatory cytokine inhibition under excessive oxidative conditions, we assessed the proinflammatory cytokine expression in H_2_O_2_-treated RBL-2H3 cells. The results showed that HPD reduced the expression of TNF-α (Figure 6h). These results suggest that HPD alleviates inflammation by decreasing intracellular oxidative stress through the regulation of antioxidant genes and the resultant inhibition of the Nrf2/HO-1 signaling pathway for mast cell activation.

### 3.7. HPD Attenuated Proinflammatory Cytokine Expression in OVA- and LPS-Induced Airway Inflammation

**Figure 7:**
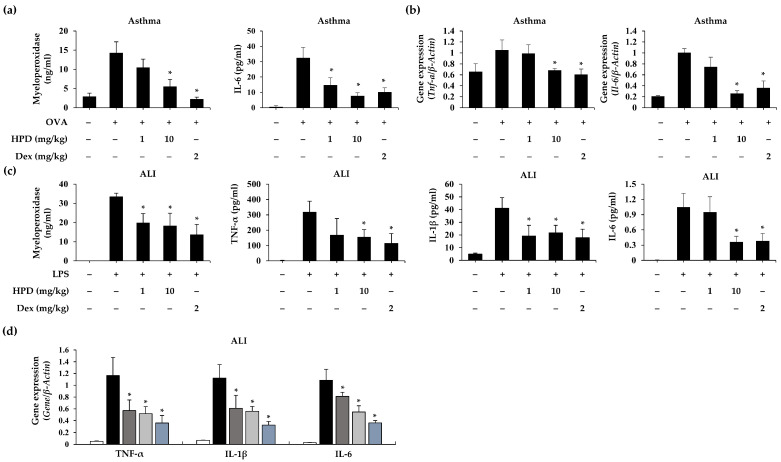
Effects of HPD on proinflammatory mediator secretion and expression in OVA and LPS-induced airway inflammation in BALF and lung homogenates. In OVA-induced allergic asthma model, (**a**) the levels of proinflammatory mediators (MPO and IL-6) were measured by ELISA and (**b**) gene expression (TNF-α and IL-6) was analyzed by qPCR. In LPS-induced lung injury model, (**c**) the levels of proinflammatory mediators (MPO, TNF-α, IL-1β and IL-6) were measured by ELISA and (**d**) gene expression (TNF-α, IL-1β, and IL-6) was analyzed by qPCR. Dex: dexamethasone. Values are mean ± SEM (*n* = 5). * *p* < 0.05 relative to OVA or ALI group.

In patients with bronchial asthma, airway inflammation phenotypes are classified based on various aspects [25]. Our OVA-induced asthma model demonstrated an increase not only the type 2 inflammatory response but also in neutrophilic and proinflammatory responses. We confirmed the production of myeloperoxidase (MPO) and IL-6 in OVA-induced mice, and HPD treatment alleviated the production of these inflammatory mediators (Figure 7a). We evaluated the gene expression of proinflammatory cytokines (TNF-α and IL-6), which increased in lungs exposed to OVA but decreased in HPD-treated lungs (Figure 7b). To investigate the efficacy of HPD on another phenotype of an airway inflammation model, the LPS-ALI model was established (Figure S2), and we measured the total number of cells in BALF to evaluate the extent of immune cell infiltration. The results showed that total cell counts were reduced by HPD treatment (Figure S4). To evaluate the HPD-induced inhibition of the inflammatory response, we assessed the secretion and expression of inflammatory mediators and proinflammatory cytokines in LPS-ALI mice using ELISA and qPCR. While the LPS-ALI group exhibited increased secretion of inflammatory mediators (MPO), and proinflammatory cytokines, including TNF-α, IL-1β, and IL-6, HPD treatment decreased the secretion of MPO and these cytokines (Figure 7c). Similar results were observed for the gene expression of proinflammatory cytokines (TNF-α, IL-1β, and IL-6) in lung tissue (Figure 7d). These results suggest that HPD attenuates inflammatory responses in heterogenous airway inflammation.

## 4. Discussion

The mechanism underlying the development of allergic airway inflammation caused by allergens is classified into two phases: sensitization and challenge [26]. In the sensitization phase, type 2 cytokines contribute to the differentiation of naïve CD4+ T cells into Th2 cells, enable specific-IgE isotype class switching of B cells, and induce the maturation and differentiation of IgE memory B cells to plasma cells that produce high levels of antigen-specific IgE [27,28,29]. This induces IgE sensitization of effector cells, such as mast cells and basophils. The challenge phase is initiated by re-exposure of allergens. The antigen-specific IgE/FcεR1 complex on the surface of sensitized mast cells and basophils reacts with the allergen to trigger the release of inflammatory mediators, including histamine, chemokines, and type 2 cytokines (IL-4, IL-5, and IL-13) [27,30,31]. The accumulation of these mediators causes bronchoconstriction, eosinophilia, recruitment of immune cells, and mucus production in inflamed tissues, ultimately resulting in allergic asthma [32]. Mast cells, as effector cells, significantly influence the pathogenesis and exacerbation of allergic asthma and affect the negative cycle of allergic asthma by contributing to Th2 cell differentiation and activation during the sensitization stage and by releasing inflammatory mediators during the challenge stage [3]. Therefore, strategically targeting mast cells in allergic asthma could be a potentially effective therapeutic strategy. Consequently, we evaluated the efficacy of HPD on mast-cell-mediated allergic airway inflammation. Moreover, we elucidated the cellular and molecular mechanisms of action of the inhibition of mast cell activation.

The findings of our current study demonstrate that HPD treatment has the potential to effectively suppress mast-cell-mediated allergic airway inflammation. To prove this theory, we used the OVA-induced allergic asthma model and DNP-HSA-stimulated RBL-2H3 cells to evaluate the effect of HPD. In our in vivo experiments, we used an OVA-induced asthma model, recognized as an eosinophilic asthma model [33]. The characteristic features of the systemic immune response in allergic asthma involve an elevated production of IgE and allergen-specific-IgE through B-cell activation, leading to splenomegaly [3]. However, our investigation revealed that HPD inhibited the systemic immune response in the OVA-induced asthma model and decreased the production of serum IgE and OVA-specific IgE. Tissue inflammation with allergen re-exposure occurs through immune cell recruitment and infiltration, with a resultant increase in the release of inflammatory mediators, including histamine, chemokines, and inflammatory cytokines [34]. In contrast, HPD not only decreased immune-cell infiltration and recruitment in OVA-exposed lung tissue but also inhibited the release of type 2 inflammatory cytokines, including IL-4, IL-5, IL-10, and IL-13. Moreover, HPD suppresses mast cell infiltration and the release of biomarkers (tryptase and β-hexosaminidase) and chemokines (CCL2, CCL3, and CXCL8) from activated mast cells [30]. In our in vitro experiments, consistent results were observed showing that HPD effectively inhibited allergic reactions, as evidenced by the reduced release of β-hexosaminidase and histamine in activated RBL-2H3 and HMC-1 cells. These mediators are known to trigger immediate-type allergic responses, such as bronchoconstriction, hypothermia, and vasodilation [35]. However, the deficiency of functional FcεR1 for HMC-1 cells alone is insufficient to study the IgE-mediated allergic reaction [36]. Thus, in our study, we used RBL-2H3 cells to investigate the mechanism of IgE-mediated degranulation. RBL-2H3 cells are commonly used for IgE-mediated degranulation due to features such as the expression of numerous IgE receptors on the cell membrane and their structural and functional similarity to mast cells [37]. During mast cell degranulation, antigen binding to the IgE–FcεR1 complex on the surface of mast cells leads to the activation of the FcεR1 signaling pathway and the resultant phosphorylation of the Src kinases Lyn, PI3K, and PLCγ [38,39], ultimately regulating the intracellular calcium ion levels and histamine release [10]. Our results showed that HPD treatment inhibited mast cell degranulation by suppressing calcium influx and inhibiting the FcεR1 signaling pathway. The activated mast cells induce an inflammatory response as well as degranulation [40]. In the activated mast cell, the signal transduction process for degranulation can induce the activation of downstream signaling molecules, including Akt [41]. The activation of Akt increases IκB phosphorylation and induces NF-κB translocation, thereby influencing the expression of various genes involved in the modulation of inflammatory responses [42,43]. In our study, DNP-HSA-stimulated RBL-2H3 cells exhibited increased expression of inflammatory cytokines and elevated NF-κB and IKKαβ phosphorylation, along with a decrease in total IκB. Notably, these effects were inhibited by HPD treatment.

Various allergic diseases induce oxidative stress, and within the context of allergic disorders, numerous reports suggest that oxidative stress considerably affects the pathogenesis of asthma [13]. In allergic asthma, the allergen indirectly promotes ROS production in the lungs through immune cell activation [44]. Deficiencies of the antioxidant defense systems lead to abnormal ROS accumulation, and a consequent increase in oxidative stress [45]. The first line of antioxidant defenses is an enzymatic system, which includes SOD, CAT, and GPx [46,47], which are biomarkers of oxidative stress [48]. In our study, DNP-HSA stimulation decreased the gene expression of SOD2 and CAT in RBL-2H3 cells, consistent with similar findings reported in patients with asthma [49,50]. However, HPD treatment restored the gene expression of SOD2 and CAT and decreased intracellular ROS levels. This result suggests that allergen exposure damages the antioxidant system, with resultant ROS accumulation, whereas HPD treatment alleviates oxidative stress by preventing damage to the antioxidant system. Nrf2, a transcription factor regulating redox homeostasis and antioxidant cellular response, is another biomarker in the antioxidant system [51]. IgE-mediated allergic reactions can trigger Nrf2 activation, leading to the expression of Heme-oxygenase-1 (HO-1) [52], a protective antioxidant enzyme [53]. The Nrf2/HO-1 signaling pathway is activated in response to excessive oxidative stress to serve as a defense mechanism against cellular stimulation [54]. Our results showed that HPD inhibited Nrf2 nuclear translocation and HO-1 expression. These findings may suggest that HPD inhibits the activation of the Nrf2/HO-1 pathway by reducing ROS accumulation through the modulation of antioxidant enzymes, including SOD2 and CAT. Furthermore, HPD reduced H_2_O_2_-induced dysfunction of antioxidant enzymes and the expression of proinflammatory cytokines, such as TNF-α. This result suggests that HPD not only inhibits allergic reactions but also hinders the development of secondary inflammatory conditions caused by oxidative stress.

Allergic asthma is generally classified as an eosinophilic airway disorder, and previous studies have focused on the role of Th2 cells and type 2 cytokines, including IL-4, IL-5, and IL-13. However, severe asthma is frequently accompanied by neutrophilic and eosinophilic inflammation [55,56]. Additionally, in the pathogenesis of asthma, oxidative stress aggravates airway inflammation by inducing proinflammatory mediators that cause severe asthma [18]. Therefore, suppression of inflammation is crucial in the management of asthma. In our studies, the OVA-induced mouse model demonstrated the release of proinflammatory cytokines and MPO, which are inflammatory biomarkers [57]. However, these inflammatory patterns were alleviated by HPD. In addition, we used the LPS-induced ALI model to prove the anti-inflammatory efficacy of HPD in the lung. This model has been previously employed in studies focusing on severe airway inflammation [58,59]. In our study, HPD treatment reduced the expression of proinflammatory mediators, including TNF-α, IL-1β, IL-6, and MPO, in LPS-induced ALI. Based on these results, we suggest that HPD may influence the development of severe asthma by mitigating persistent allergic airway inflammation caused by allergens and oxidative stress.

## 5. Conclusions

In conclusion, HPD treatment effectively mitigated mast-cell-mediated allergic airway inflammation by alleviating mast cell degranulation through inhibition of the degranulation pathway and intracellular calcium influx. HPD also reduced inflammatory cytokine expression by suppressing NF-κB phosphorylation in DNP-HSA-stimulated RBL-2H3 cells. HPD alleviated oxidative stress in both DNP-HSA- and H_2_O_2_-stimulated RBL-2H3 cells, resulting in the regulation of the Nrf2/HO-1 pathway. These therapeutic effects of HPD were demonstrated in both the OVA-induced allergic asthma and LPS-induced lung injury models. Therefore, based on our findings, we suggest that HPD could serve as a potential therapeutic agent for the management of allergic airway inflammatory diseases.

## Data Availability

The data shown in this article are available from the corresponding authors upon a reasonable request.

## Supplementary Materials

The following supporting information can be downloaded at: https://www.mdpi.com/article/10.3390/antiox13050528/s1, Table S1: The primer sequences for the qPCR; Table S2: The list of antibodies; Figure S1: OVA-induced allergic asthma model; Figure S2: LPS-induced acute lung injury model; Figure S3: Effects of HPD on continuous calcium measurements; Figure S4: Effects of HPD on cellular infiltration into BALF.
