# Supplementary material for: Hispidulin Alleviates Mast Cell-Mediated Allergic Airway Inflammation through FcεR1 and Nrf2/HO-1 Signaling Pathway
Source: Antioxidants (Basel). 2024 Apr 26;13(5):528. doi: 10.3390/antiox13050528 (PMC11118000; doi:10.3390/antiox13050528)
Supplement: Supplementary file 1 [file antioxidants-13-00528-s001.zip › antioxidants-2952884-supplementary.pdf]

Supporting information

Hispidulin alleviates mast cell-mediated allergic airway inflammation through FcεR1 and Nrf2/HO-1 signaling pathway

Seungwon Jeong [1,2], Yeon-Yong Kim [1], Dongwon Lee [2,3], Sang-Hyun Kim [4,], Soyoung Lee [1,*]*

[1] Functional Biomaterial Research Center, Korea Research Institute of Bioscience and Biotechnology (KRIBB), 181, Ipsin-gil, Jeongeup 56212, Republic of Korea

[2] Department of Bionanotechnology and Bioconvergence Engineering, Jeonbuk National University, 567, Baekje-daero, Jeonju 54896, Jeonbuk, Republic of Korea

[3] Department of Polymer Nano Science and Technology, Jeonbuk National University, 567, Baekje-daero, Jeonju 54896, Jeonbuk, Republic of Korea

[4] CMRI, Department of Pharmacology, School of Medicine, Kyungpook National University, Daegu 41944, Republic of Korea

Table S1. The primer sequences for the qPCR

RAT primer				
**No.**	**Name**	**Source**	**Forward(Sense)**	**Reverse(Antisense)**
1	Rat IL-4	RAT	ACC TTG CTG TCA CCC TGT TC	TTG TGA GCG TGG ACT CAT TC
2	Rat IL-6	RAT	TGT GCA ATG GCA ATT CTG AT	GAG CAT TGG AAG TTG GGG TA
3	Rat IL-13	RAT	CCA CAG GAC CCA GAG GAT ATT GA	TAG CGG AAA AGT TGC TTG GAG TAA
4	Rat TNF-α	RAT	TCC CAA ATG GGC TCC CTC TC	AAA TGG CAA ACC GGC TGA CG
5	Rat SOD2	RAT	AGC TGC ACC ACA GCA AGC AC	TCC ACC ACC CTT AGG GCT CA
6	Rat CAT	RAT	TCC GGG ATC TTT TTA ACG CCA TTG	TCG AGC ACG GTA GGG ACA GTT CAC
7	Rat GPx	RAT	CGG TTT CCC GTG CAA TCA GT	ACA CCG GGG ACC AAA TGA TG
8	Rat β-actin	RAT	GAA GCT GTG CTA TGT TGC CCT AGA	GTA CTC CTG CTT GCT GAT CCA CAT

Mouse primer				
**No.**	**Name**	**Source**	**Forward(Sense)**	**Reverse(Antisense)**
1	M IL-1β	Mouse	GGC CGA CTT CAC TGT ACA AC	CCT TCA CAG AGA GGG TCA CAG
2	M IL-4	Mouse	TCT CGA ATG TAC CAG GAG CCA TAT C	AGC ACC TTG GAA GCC CTA CAG A
3	M IL-5	Mouse	ATG GAG ATT CCC ATG AGC AC	AGC CCC TGA AAG ATT TCT CC
4	M IL-6	Mouse	CCA CTT CAC AAG TCG GAG GCT TA	GCA AGT GCA TCA TCG TTG TTC ATA C
5	M IL-10	Mouse	TGG CCC AGA AAT CAA GGA GC	CAG CAG ACT CAA TAC ACA CT
6	M IL-13	Mouse	CCT CTG ACC CTT AAG GAG CTT	ATG TTG GTC AGG GAA TCC AG
7	M TNF-α	Mouse	AAG CCT GTA GCC CAC GTC GTA	GGC ACC ACT AGT TGG TTG TCT TTG
8	M CCL2	Mouse	CAG CAG GTG TCC CAA AGA A	CTT GAG GTG GTT GTG GAA AA
9	M CCL3	Mouse	TCT GCA ACC AAG TCT TCT CAG	GAA GAG TCC CTC GAT GTG GAT A
10	M CXCL8	Mouse	CCA CCT TGA GGC AAG ATC CC	CCC AGA ATC AAC GCA AAG CC
11	M Tpsg1	Mouse	GGT CAC ACT GTC TCC CCA CT	GCA TCC CAG GGT AGA AGT CA
12	M c-Kit	Mouse	AGC AAT GGC CTC ACG AGT TCT A	CCA GGA AAA GTT TGG CAG GAT
13	M β-Actin	Mouse	TAG ACT TCG AGC AGG AGA TG	TTG ATC TTC ATG GTG CTA GG

Table S2. The list of antibodies

Antibody				
Protein	Type of antibody	Cat.	Dilution	Supplier
Phospho-Akt (Ser473)	Rabbit polyclonal	9271	1/1000	CST
Phospho-IKKαβ (Ser176/180)	Rabbit monoclona	2697	1/1000	CST
Phospho-Lyn (Tyr507)	Rabbit polyclonal	2731	1/1000	CST
Phospho-PI3K p85	Rabbit polyclonal	4228	1/1000	CST
Phospho-PLC-γ (Tyr783)	Rabbit monoclona	2821	1/1000	CST
β-Actin	Rabbit monoclona	4967	1/1000	CST
Akt	Rabbit polyclonal	9272	1/1000	CST
HO-1	Rabbit monoclona	5853	1/1000	CST
IκBα	Rabbit monoclona	4812	1/1000	CST
Lamin B1	Rabbit monoclona	12586	1/1000	CST
Lyn	Rabbit polyclonal	2732	1/1000	CST
Nrf2	Rabbit monoclona	12721	1/1000	CST
PI3K p85	Rabbit polyclonal	4292	1/1000	CST
PLC-γ	Rabbit monoclona	2822	1/1000	CST

Figure S1. OVA-induced allergic asthma model

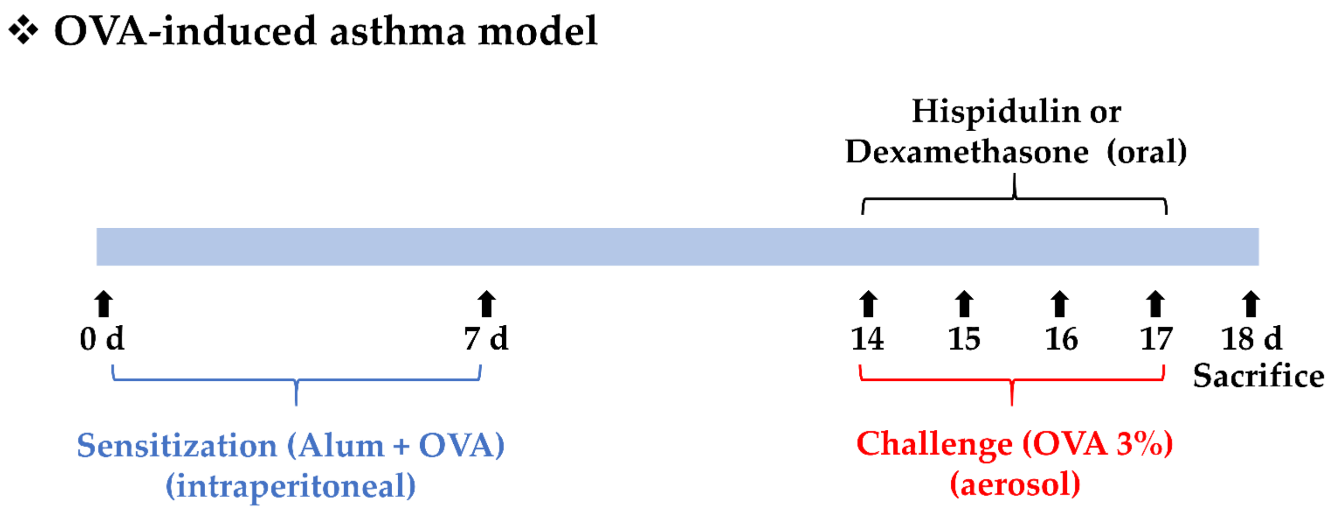

Figure S2. LPS-induced acute lung injury model

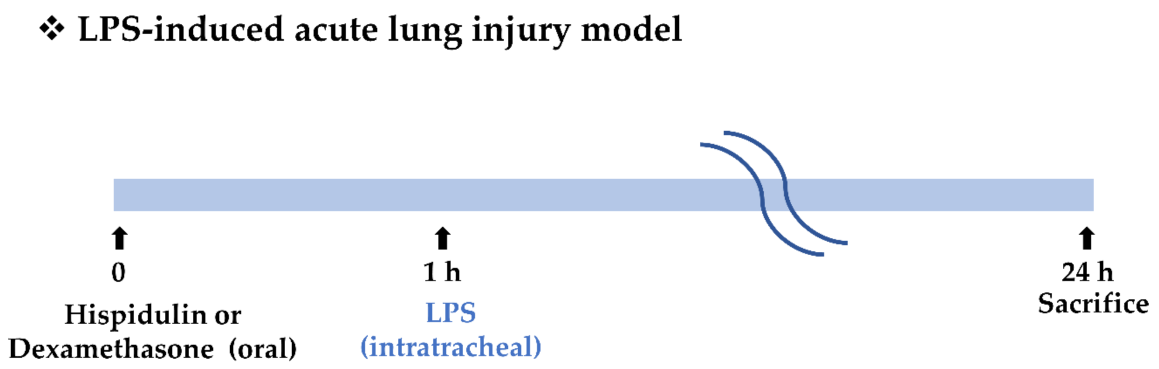

Figure S3. Effects of HPD on continuous calcium measurements

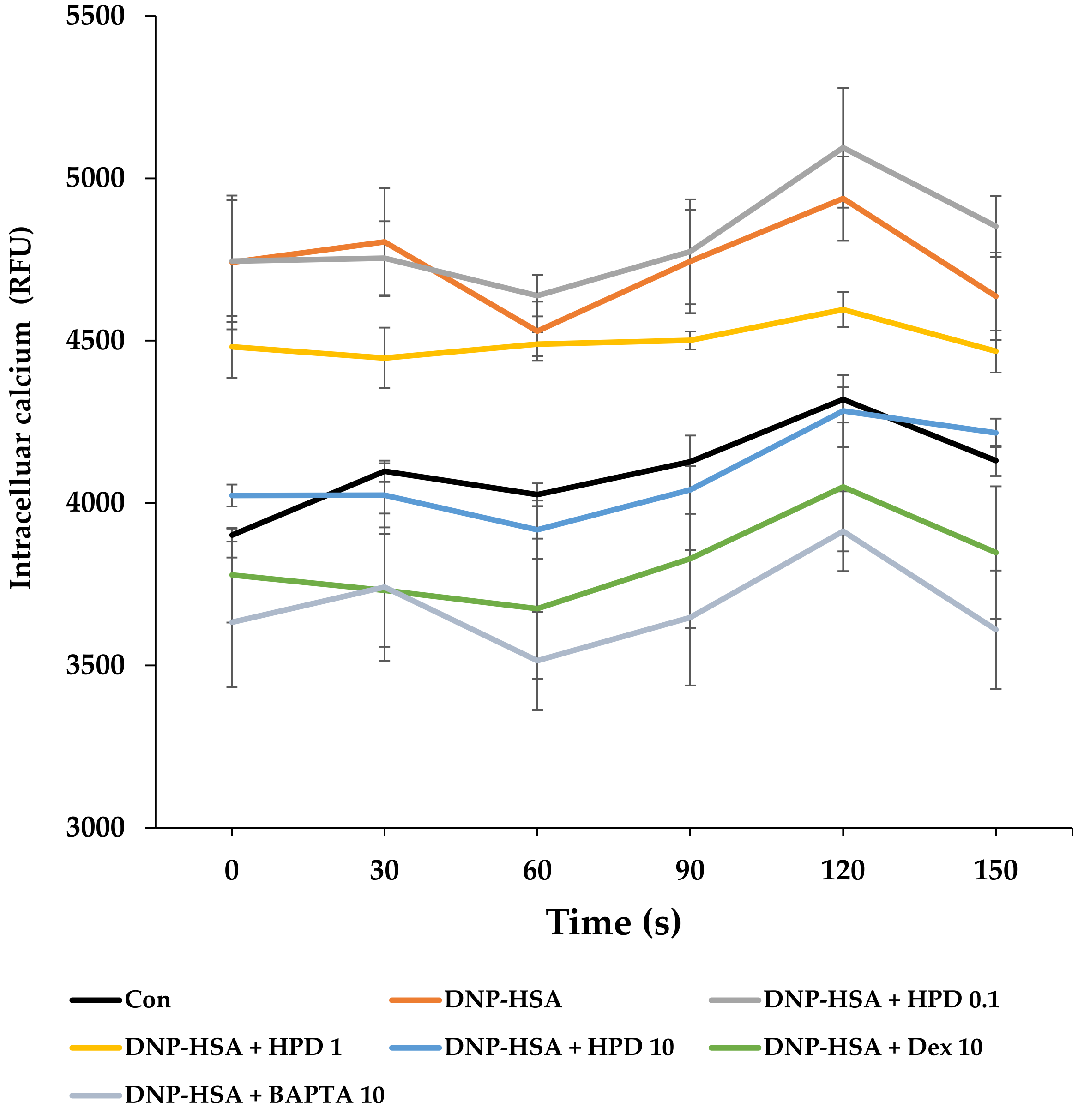

The intracellular calcium level was kinetically measured for total 150 s, every 30 s, 5 times. Values are mean ± SEM (n=5). *p < 0.05 relative to DNP-HSA group.

Figure S4. Effects of HPD on cellular infiltration into BALF

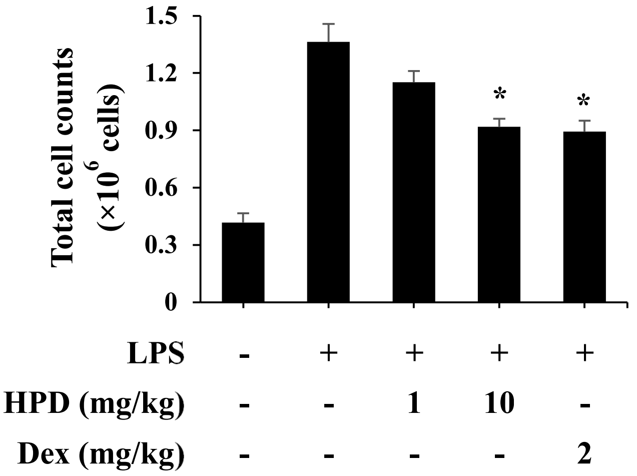

The variation of total cell counts in BALF of LPS-induced ALI model. Values are mean ± SEM (n=5). *$p < 0.05$ relative to LPS group.
